# Isorhamnetin Promotes 53BP1 Recruitment through the Enhancement of ATM Phosphorylation and Protects Mice from Radiation Gastrointestinal Syndrome

**DOI:** 10.3390/genes12101514

**Published:** 2021-09-26

**Authors:** Yuichi Nishiyama, Akinori Morita, Shogo Tatsuta, Misaki Kanamaru, Masahiro Sakaue, Kenta Ueda, Manami Shono, Rie Fujita, Bing Wang, Yoshio Hosoi, Shin Aoki, Takeshi Sugai

**Affiliations:** 1 Graduate School of Biomedical Sciences, Tokushima University, Tokushima 770-8503, Japan; y-nishi@tokushima-u.ac.jp (Y.N.); aghostu99652100@gmail.com (S.T.); marumarumarumi326@gmail.com (M.K.); sakamasa135@gmail.com (M.S.); shirumi2828@gmail.com (K.U.); shomanami1215@gmail.com (M.S.); 2 Faculty of Pharmacy, Keio University, Tokyo 105-8512, Japan; krnfujita@z8.keio.jp (R.F.); sugai-tk@pha.keio.ac.jp (T.S.); 3 National Institute of Radiological Sciences, National Institutes for Quantum and Radiological Science and Technology, Chiba 263-8555, Japan; wang.bing@qst.go.jp; 4 Department of Radiation Biology, Graduate School of Medicine, Tohoku University, Sendai 980-8575, Japan; hosoi@med.tohoku.ac.jp; 5 Faculty of Pharmaceutical Sciences, Tokyo University of Science, Chiba 278-8510, Japan; shinaoki@rs.noda.tus.ac.jp

**Keywords:** isorhamnetin, radiation hematopoietic syndrome, radiation gastrointestinal syndrome, DNA damage response, p53, p53 target genes, 53BP1, ATM, pS1981-ATM, γH2AX

## Abstract

Flavonoids are a subclass of polyphenols which are attractive, due to possessing various physiological activities, including a radioprotective effect. Tumor suppressor p53 is a primary regulator in the radiation response and is involved in the pathogenesis of radiation injuries. In this study, we revealed that isorhamnetin inhibited radiation cell death, and investigated its action mechanism focusing on DNA damage response. Although isorhamnetin moderated p53 activity, it promoted phosphorylation of ataxia telangiectasia mutated (ATM) and enhanced 53BP1 recruitment in irradiated cells. The radioprotective effect of isorhamnetin was not observed in the presence of ATM inhibitor, indicating that its protective effect was dependent on ATM. Furthermore, isorhamnetin-treated mice survived gastrointestinal death caused by a lethal dose of abdominal irradiation. These findings suggested that isorhamnetin enhances the ATM-dependent DNA repair process, which is presumably associated with the suppressive effect against GI syndrome.

## 1. Introduction

Preventing hematopoietic (HP) and gastrointestinal (GI) syndromes after high-dose radiation exposure is important for reducing mortality. In particular, radiation GI syndrome is incurable because effective therapy for the syndrome has not established yet. Development of effective radioprotector or radiomitigator is an essential provision for nuclear terrorism, radiation disaster, and planned exposure such as radiotherapy.

Tumor suppressor p53 is a primary regulator in radiation response and is well known as a transcription factor which is closely associated with apoptosis [1], cell cycle control [2], and DNA damage response [3]. Several studies demonstrated that p53 functions in a different way between tissues [4,5,6]. p53 causes apoptosis in irradiated hematopoietic lineages that leads to HP syndrome, and some p53 inhibitors have been shown to rescue mice from HP syndrome caused by total-body irradiation (TBI) [7,8,9]. On the other hand, p53 acts as a radioresistant factor against radiation GI injury [4,5]. We focused on the different roles in the tissues and identified some p53-regulating compounds [9,10,11]. Among them, 5-chloro-8-quinolinol (5CHQ) rescues mice from abdominal irradiation-induced GI syndrome through the radioprotective modulation of p53 transactivation [11]. p53 regulation is considered as one of the beneficial counter strategies against radiation toxicity.

Flavonoids are a subclass of polyphenols and is attractive due to possessing various physiological activities, such as tumor suppressing [12] and antioxidative [13] effects. In terms of the radioprotective effect, some flavonoids have been shown to suppress radiation HP and GI toxicity. Apigenin, a rich element found in dark-green vegetables, has been shown to suppress TBI-induced HP [14] and GI toxicities in mice [15]. Another report demonstrated that alcohol-extracted flavonoid inhibits hemocyte reduction after TBI [16]. However, there was no report on the p53-dependent radioprotective effect of flavonoids.

In this study, we performed a screening of flavonoids, in order to specify novel radioprotective ones, and selected isorhamnetin as a candidate. Isorhamnetin moderately inhibited p53 activity, but promoted ATM activation and the recruitment of DNA repair factor in irradiated cells. Moreover, it effectively suppressed GI death after a lethal dose of abdominal irradiation.

## 2. Materials and Methods

### 2.1. Cell Culture and Treatment

The human T-cell leukemia MOLT-4 cell line bearing wild-type p53 [17,18] was established and gifted from Dr. Jun Minowada (Roswell Park Memorial Institute, Buffalo, NY, USA), and their derivative transformed cell lines (KD-1 (p53-knockdown by p53-targeting shRNA), Nega (negative control of the shRNA), and KD-1/R-p53-1 (p53-revertant of KD-1)) were established as described in our previous reports [19,20]. They were cultured in RPMI 1640 medium (Wako, Osaka, Japan). The human hepatoblastoma cell line Hep G2 cells [21] purchased from ATCC (HB-8065) were cultured in DMEM/F12 medium (Wako). The media were supplemented with 10% fetal bovine serum (FBS) (Gibco, CA, USA) and antibiotics (Nacalai Tesque, Kyoto, Japan) including 100 U/mL penicillin and 0.1 mg/mL streptomycin. They were maintained at 37 °C in a humidified atmosphere with 5% CO_2_ and irradiated using an X-ray generator (MBR-1520R-3, Hitachi, Tokyo, Japan) under a tube voltage of 150 kV, a tube current of 20 mA, and a dose rate of approximately 1.6 Gy/min. Dosimetry was carried out with a 0.3 cc N31003 ionization chamber (PTW Freiburg, Freiburg, Germany).

### 2.2. Cell Survival Assays

Radioprotective flavonoids were screened by WST-8 assay using MOLT-4 cells. The cells were treated with 18 kinds of flavonoids, kindly gifted by Prof. Yoshichika Kawai (Graduate School of Biomedical Sciences, Tokushima University), or vehicle (dimethyl sulfoxide: DMSO, Wako) an hour before irradiation. After twenty-four hours of 10 Gy irradiation, cell viabilities were evaluated by using a Cell Counting Kit-8 (DOJINDO, Kumamoto, Japan), and isorhamnetin was selected as a candidate of radioprotective flavonoid. Absorbance was measured at 450 nm using a spectrophotometer (Thermo Fisher Scientific, Waltham, MA, USA).

Dye exclusion test was performed for further assessment of the anti-cell death activity of isorhamnetin. MOLT-4 cells and their transfectants were treated with isorhamnetin or DMSO, incubated for an hour, and irradiated with 10 Gy irradiation. Living and dead cells were counted differently by staining with erythrosine B (Tokyo kasei, Tokyo, Japan), and cell viabilities were evaluated at 24 h after the irradiation.

### 2.3. Apoptosis Assay

Antiapoptotic effect of isorhamnetin was investigated by Annexin V-FITC staining. MOLT-4 cells and their transfectants were treated with 20 μM isorhamnetin or DMSO an hour before irradiation. After twenty hours of 10 Gy irradiation, the percentages of cells in apoptosis were examined by a flow cytometer (FACS Calibur, Becton Dickinson, NJ, USA) using an MEBCYTO Apoptosis kit (MBL, Tokyo, Japan)

### 2.4. Colony Formation Assay

MOLT-4 cells were treated with isorhamnetin or DMSO with or without 20 μM KU55933 (Synkinase, Shanghai, China), a specific inhibitor of ATM kinase, an hour before irradiation. After twenty-four hours of 2 Gy irradiation, cells were diluted and plated in 4 mL of 0.16% soft agar medium at a density of 150–6000 cells/25 cm^2^ dish, followed by incubation for 3 weeks. Surviving fraction was calculated by counting spheroids consisting of more than 50 cells.

### 2.5. Immunoblotting

MOLT-4 cells were treated with 40 μM isorhamnetin or DMSO with or without 20 μM KU55933 an hour before irradiation. After 0.5, 3, and 6 h of 10 Gy irradiation, cells were collected and prepared as samples. Protein concentrations of the samples were adjusted equally using a BCA Protein Assay Reagent (Thermo Fisher Scientific). SDS-PAGE was conducted using 5–20% gradient pre-cast gels (ATTO, Tokyo, Japan). Western blotting was performed using the following antibodies: H2AX (D17A3, Cell Signaling Technology, Danfoss, MA, USA), γH2AX (JBW301, Millipore, MA, USA), ATM (NB100-104, Novus Biologicals, Littleton, CO, USA), pS1981-ATM (pATM) (D6H9, Cell Signaling Technology), p53 (clone DO-1, sc-126 HRP, Santa Cruz Biotechnology, Dallas, CA, USA), pS15-p53 (9284, Cell Signaling Technology), p21 (clone EA10, Calbiochem, Kenilworth, CA, USA), PUMA (sc19187, Calbiochem), β-actin (AC-15, Sigma, St. Louis, MO, USA). β-actin was used as an internal control.

### 2.6. Immunofluorescence

Hep G2 cells were treated with 40 μM isorhamnetin or DMSO with or without 20 μM KU55933. They were fixed with 4% paraformaldehyde/phosphate-buffered saline (PBS) for 30 min at 0.5 and 4 h after 2 Gy irradiation for γH2AX and 53BP1 focus assays, respectively. The cells were permeabilized with 0.2% Triton X-100 (Wako)/PBS and blocked with 1% bovine serum albumin (Calbiochem)/PBS. Then, cells were incubated with primary antibodies overnight at 4 °C, incubated with Alexa 546-labeled goat anti-mouse IgG antibody (Life Technologies, Carlsbad, CA, USA) for 1 h at room temperature, and mounted with VECTASHIELD mounting medium with DAPI (Vector Laboratories, Burlingame, CA, USA). JBW301 (Millipore) and clone BP13 (Millipore) were used as primary antibodies for γH2AX and 53BP1, respectively. γH2AX and 53BP1 foci were observed by the BZ-9000 fluorescence microscopy (Keyence, Osaka, Japan), and the number of foci showing brightness value of 38 or more was automatically counted using a BZ analyzer software (Keyence, Osaka, Japan). The foci number per nucleus was quantified from 50 nuclei.

### 2.7. Mice

Specific pathogen-free female ICR mice (7 weeks of age) were obtained (SLC, Shizuoka, Japan), and were housed under a controlled temperature (22 °C) and a preset light-dark cycle. Standard MF diet and normal drinking water were provided ad libitum during all experimental periods. Ethics approval for the experimental design was obtained from the animal experimental committee of Tokushima University

### 2.8. Mouse Toxicity Test

After 7 days of acclimatization, mice were given a single intraperitoneal injection of isorhamnetin (7.5, 15, and 30 mg/kg body weight) or vehicle (10% DMSO in olive oil), and changes in body weight were recorded every 7 days. Mice were sacrificed 61 days after the injection, and blood was collected from the heart. The amount of red blood cells (RBC), hemoglobin (Hgb), white blood cells (WBC), and platelets (PLT) were measured using an automated impedance-based hematology analyzer (Microcemi LC-662, Horiba, Kyoto, Japan).

### 2.9. Induction of Acute Radiation Syndromes

After 7 days of acclimatization, mice were given a single intraperitoneal injection of 30 mg/kg isorhamnetin or vehicle (10% DMSO in olive oil) 30 min before irradiation. TBI and subtotal body irradiation (SBI) were then performed to induce acute HP and GI syndromes, respectively, using the X-ray generator (MBR-1520R-3, Hitachi) under a tube voltage of 150 kV, a tube current of 20 mA, and a dose rate of approximately 1.0 Gy/min. Dosimetry was carried out with a 0.3 cc N31003 ionization chamber (PTW Freiburg). The SBI treatment was conducted in a manner that permits bone marrow death to be avoided because bone marrow injury is considered as a contributing factor to GI death [22]. Briefly, the frontal side of the body, including head, chest, and front legs, were shielded with lead layers with a thicknesses of 3 cm, and X-irradiation was then performed in a dorsal-to-ventral direction.

### 2.10. Statistical Analyses

Data are presented as the mean ± standard deviation. Statistical differences between groups were examined using chi-square test for mouse survival data. Student’s t-test was used for data of immunoblotting, apoptosis assay, and dye exclusion test using derivative transformed cell lines of MOLT-4 cells. Mann–Whitney U test was used for γH2AX and 53BP1 foci data. Dunnett’s test was used for data of colony formation assay, WST-8 assay, dye exclusion test without using transfectants of MOLT-4 cells, and hematology. *p* < 0.05 was considered significant.

## 3. Results

### 3.1. Isorhamnetin Inhibits Radiation Cell Death

**Figure 1:**
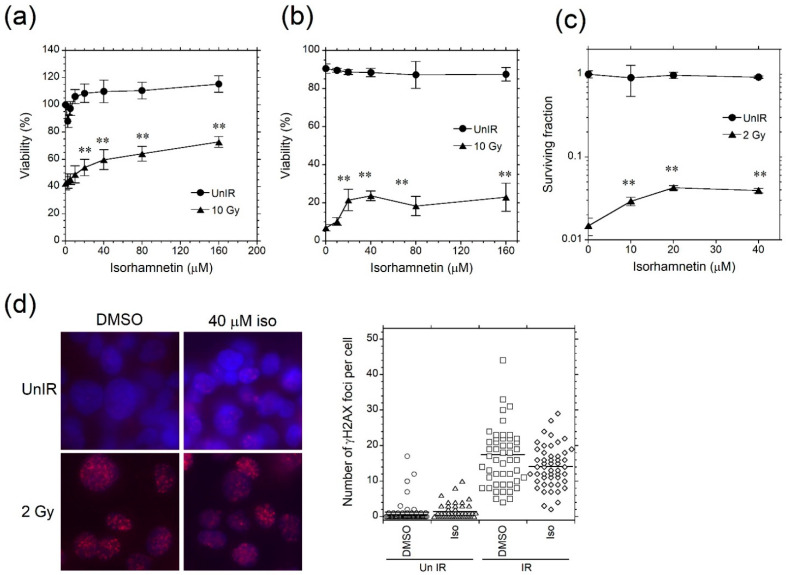
Isorhamnetin inhibits radiation-induced cell death. (**a**) WST-8 assay of MOLT-4 cell viability 24 h after 10 Gy irradiation (*n* = 3). (**b**) Dye exclusion test of MOLT-4 cells 24 h after 10 Gy irradiation (*n* = 3). (**c**) Colony forming activity of MOLT-4 cells treated with 2 Gy irradiation (*n* = 5). (**d**) γH2AX foci in Hep G2 cells 30 min after 2 Gy irradiation. The foci and DNA were stained with γH2AX-specific antibody and DAPI, respectively. The number of γH2AX foci per nucleus was quantified (*n* = 50). Magnification, 40×. Data represent the mean ± standard deviation (**a**–**c**). Asterisk indicates a significant difference (** *p* < 0.01) between groups. *DMSO* dimethyl sulfoxide, *Iso* isorhamnetin, *IR* irradiated, *UnIR* unirradiated.

Candidates for high radioprotective flavonoid were screened by WST-8 assays in a blind manner (Supplementary Figure S1). Among 18 kinds of flavonoids, isorhamnetin was found as a radioprotective and low cytotoxic one. Isorhamnetin showed a dose-dependent radioprotective effect in the range of 20–160 μM, while unirradiated MOLT-4 cells showed more than 100% viability with isorhamnetin treatment above 10 μM. This may be attributed to the self-absorption of the 450 nm wavelength by isorhamnetin. The dye exclusion test and the colony formation assay also revealed a significant and dose-dependent radioprotective effect of isorhamnetin in the range of 20–160 µM and 10–40 μM, respectively (Figure 1b,c).

In the WST-8 assay, apigenin also exhibited significant radioprotective activity in the range from 12.5 to 40 μM, and its anti-cell death effect was comparable to that of isorhamnetin (Supplementary Figure S1). However, its cytotoxicity was more potent compared with that of isorhamnetin. Apigenin has already been shown to protect mice from TBI-induced HP and GI toxicities [14,15], and the present study demonstrated that it had no efficacy against GI death caused by SBI (Supplementary Figure S2). Therefore, isorhamnetin was selected as a novel radioprotective flavonoid and was proceeded with detailed investigations.

For further assessment of the radioprotective effect, γH2AX foci in irradiated cells treated with or without isorhamnetin were investigated (Figure 1c). There were no significant differences in the number of γH2AX foci between isorhamnetin-treated cells and DMSO-treated cells at 30 min after 2 Gy irradiation, indicating that initial radiation DNA damage was at a similar level between them. These findings also suggested that isorhamnetin had no radical scavenging activity.

### 3.2. Isorhamnetin Inhibits Radiation Cell Death Independent of p53-Mediated Apoptosis

**Figure 2:**
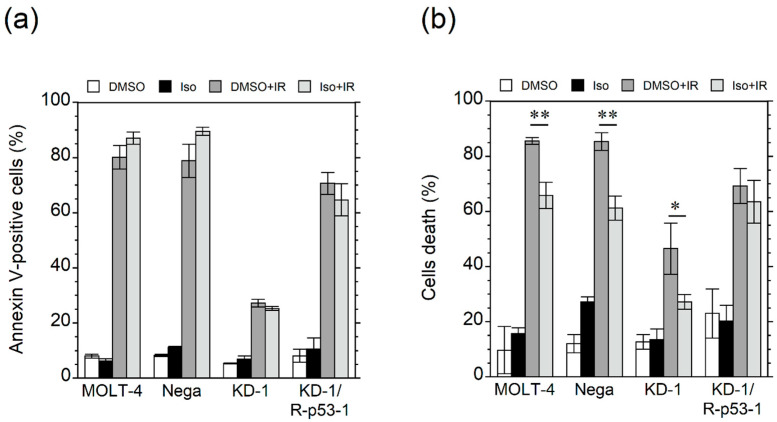
Isorhamnetin inhibits radiation cell death independent of p53 status and apoptosis. Cells were treated with 20 μM isorhamnetin. (**a**) Apoptosis assay by Annexin V-FITC staining 20 h after 10 Gy irradiation (*n* = 3). (**b**) Dye exclusion test 24 h after 10 Gy irradiation (*n* = 3). Asterisks indicate a significant difference (* *p* < 0.05 and ** *p* < 0.01) between groups. *DMSO* dimethyl sulfoxide, *Iso* isorhamnetin, *IR* irradiated.

Antiapoptotic effect of isorhamnetin was examined by Annexin V-FITC staining. As shown in Figure 2a, isorhamnetin did not suppress radiation-induced apoptosis in MOLT-4 cells and their transfectants. However, significant reductions in cell death were observed in MOLT-4 cells and their transfectants except KD-1/R-p53-1 in the dye exclusion test (Figure 2b). These findings indicated that isorhamnetin enhanced cell viability after irradiation independent of p53 status and p53-mediated apoptosis.

### 3.3. Isorhamnetin Protects Mice from SBI-Induced GI Death

**Figure 3:**
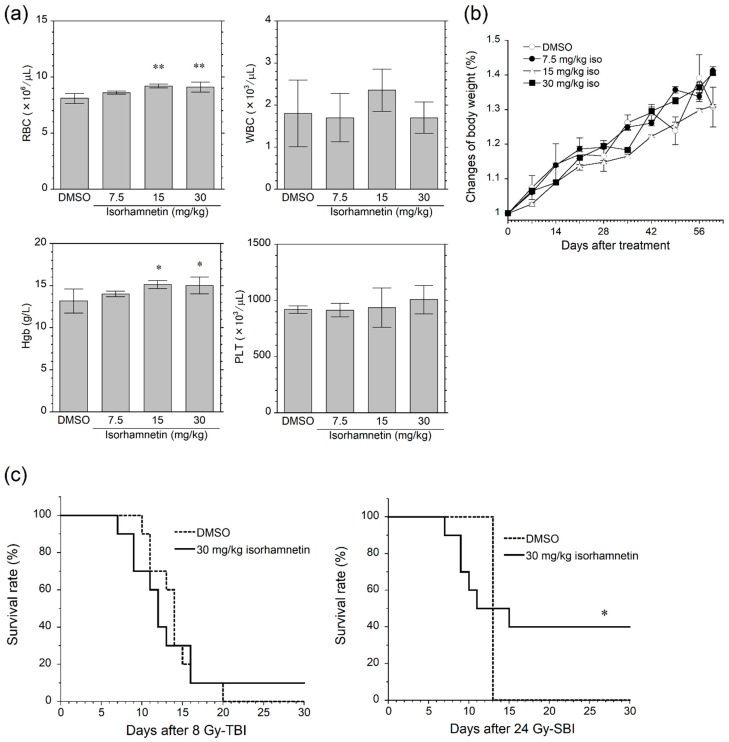
Isorhamnetin rescues mice from gastrointestinal death after SBI, but not from bone marrow death after TBI. (**a**) Hematological assay 61 days after single-dose of isorhamnetin (*n* = 4 in each group). (**b**) Body weight changes after the isorhamnetin treatment (*n* = 4 in each group). (**c**) 30-day survival tests after 8 Gy TBI and 24 Gy SBI (*n* = 10 in each group). Data represent the mean ± standard deviation. Asterisks indicate a significant difference (* *p* < 0.05 and ** *p* < 0.01) between groups. *DMSO* dimethyl sulfoxide, *Iso* isorhamnetin, *RBC* red blood cells, *Hgb* hemoglobin, *WBC* white blood cells, *PLT* platelets, *TBI* total body irradiation, *SBI* subtotal body irradiation.

The acceptable dose of isorhamnetin to mice was investigated preliminarily. Although RBC and Hgb levels in mice treated with 7.5 mg/kg and 30 mg/kg isorhamnetin were significantly higher than those in mice treated with DMSO on day 61 after the treatment, these differences were exceedingly small (Figure 3a). Moreover, there were no abnormalities in weight gain at each dose tested (Figure 3b). Based on these findings, the dose to mice was thought to be allowed at least 30 mg/kg or less.

The radioprotective effect of isorhamnetin was investigated in mouse models of acute radiation syndromes. Radiation HP and GI syndromes were induced by 10 Gy TBI and 24 Gy SBI, respectively. Isorhamnetin significantly improved survival rate of mice after the SBI treatment but failed to rescue mice after the TBI treatment (Figure 3c). These results suggested that isorhamnetin enhanced radioresistance of the intestinal tissue but not the myeloid tissue.

### 3.4. Isorhamnetin Promotes Phosphorylation of ATM after Irradiation

**Figure 4:**
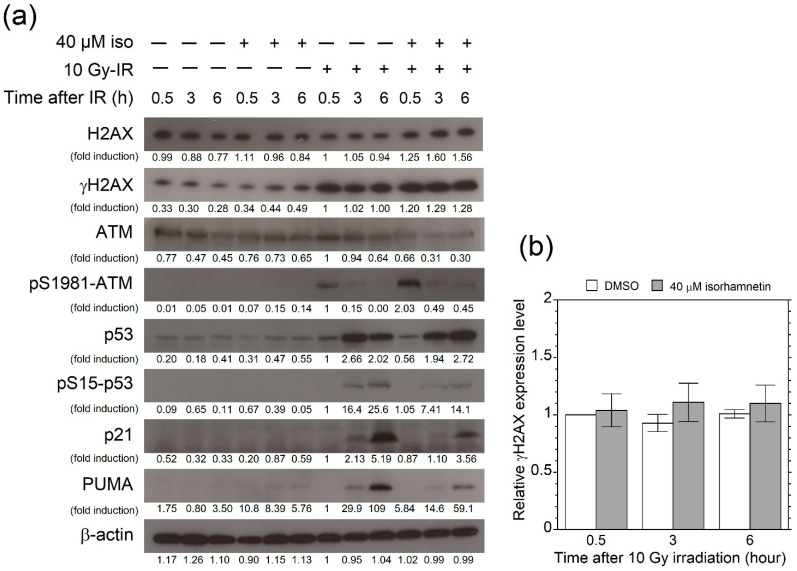
Isorhamnetin enhances ATM phosphorylation in irradiated MOLT-4 cells. (**a**) DNA damage response-related gene products were detected by Western blotting 0.5, 3, and 6 h after 10 Gy irradiation, and signal intensity of each band was analyzed by densitometry. The original value of each signal intensity ratio was determined using the signal intensity obtained from cells 0.5 h after 10 Gy irradiation (1.5 h after DMSO treatment) as a control (lane 7). The band intensity ratio of each sample was then normalized by the band intensity ratio of β-actin as an internal control. (**b**) Relative expression level of γH2AX (*n* = 3). Data represent the mean ± standard deviation. *DMSO* dimethyl sulfoxide, *Iso* isorhamnetin, *IR* irradiated.

Expression of radiation-induced DNA damage response-related proteins in MOLT-4 cells were investigated by Western blot analyses (Figure 4a). p53 and its target genes (p21, PUMA) were strongly induced by 10 Gy irradiation, and isorhamnetin slightly enhanced p53 stability at 6 h after irradiation. However, at 3 and 6 h after irradiation, isorhamnetin decreased phosphorylated p53 (pS15-p53), p21, and PUMA expressions in irradiated cells by nearly half.

Although isorhamnetin did not affect the amount of initial radiation DNA damage (Figure 1d), we observed an increased expression of γH2AX in isorhamnetin-treated cells compared with vehicle-treated cells at 0.5, 3, and 6 h after the irradiation. The increasing tendency in the γH2AX expression was obtained in three independent experiments, but statistical significance was not detectable (Figure 4b). Furthermore, ATM phosphorylation (pS1981-ATM) was strongly induced in isorhamnetin-treated cells at 0.5 h after the irradiation. These findings suggested that isorhamnetin enhanced ATM-mediated DNA damage response.

### 3.5. Isorhamnetin Promotes 53BP1 Recruitment through the Enhancement of ATM Phosphorylation

**Figure 5:**
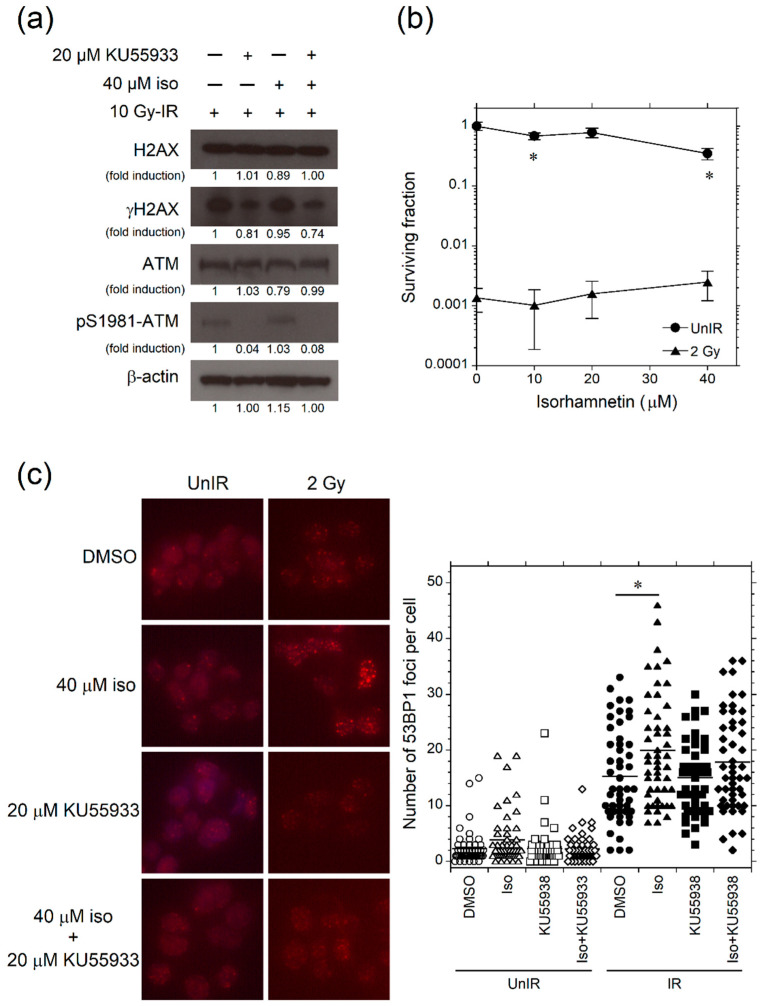
Isorhamnetin exhibits radioprotective effect through the ATM-dependent promotion of 53BP1 recruitment. (**a**) Isorhamnetin could not promote ATM and H2AX phosphorylation in irradiated MOLT-4 cells at the presence of ATM inhibitor (KU55933). Data were obtained 0.5 h after 10 Gy irradiation. The original value of each signal intensity ratio was determined using the signal intensity obtained from cells 0.5 h after 10 Gy irradiation (1.5 h after DMSO treatment) as a control (lane 1). The band intensity ratio of each sample was then normalized by the band intensity ratio of β-actin as an internal control. (**b**) Isorhamnetin could not enhance colony forming activity in the presence of the ATM inhibitor (*n* = 5). (**c**) 53BP1 foci in Hep G2 cells 4 h after 2 Gy irradiation. The foci and DNA were stained with 53BP1-specific antibody and DAPI, respectively. The number of 53BP1 foci per nucleus was quantified (*n* = 50). Magnification, 40×. An asterisk indicates a significant difference (* *p* < 0.05) between groups. *DMSO* dimethyl sulfoxide, *Iso* isorhamnetin, *IR* irradiated, *UnIR* unirradiated.

The findings shown in Figure 4 lead to the hypothesis that ATM is associated with the radioprotective effect of isorhamnetin. We next investigated whether the radioprotective effect of isorhamnetin depends on ATM using the ATM inhibitor KU55933. As shown in Figure 5a, KU55933 inhibited pS1981-ATM and γH2AX expression in isorhamnetin-treated MOLT-4 cells. Moreover, in contrast to the finding from Figure 1c, isorhamnetin could not elevate colony surviving fraction following 2 Gy irradiation in the presence of KU55933 (Figure 5b). These findings indicated that ATM has a crucial role in the radioprotective effect of isorhamnetin.

To examine the influence of isorhamnetin on the radiation DNA damage repair pathway, we investigated 53BP1 foci formation in irradiated Hep G2 cells. The findings from Figure 1 demonstrated that isorhamnetin had no effect on inhibiting initial radiation DNA damage. However, compared with vehicle-treated cells, isorhamnetin-treated cells showed a significant increase in the number of 53BP1 foci at 4 h after 2 Gy irradiation, and the increase was not observed in the presence of KU55933 (Figure 5c). These findings suggested that isorhamnetin promotes radiation DNA damage repair through the enhancement of ATM-dependent 53BP1 recruitment.

## 4. Discussion

The findings reported herein show that isorhamnetin has an ATM-dependent radioprotective effect through the promotion of 53BP1 recruitment. Isorhamnetin was found as a radioprotective flavonoid that effectively suppressed radiation GI death. It inhibited radiation cell death independent of p53 status without radical scavenging and antiapoptotic activities. Its protective effect may be attributed to the enhancement of an ATM-dependent DNA damage repair pathway.

We found that isorhamnetin has a radioprotective activity with low cytotoxicity. While isorhamnetin showed no antiapoptotic effect, it inhibited radiation cell death independent of p53 status. The anti-cell death effect is likely attributed to the inhibition of non-apoptotic cell death, and use of isorhamnetin may not be beneficial for suppressing radiation injury mainly caused by apoptosis. The finding from 30-day survival tests that isorhamnetin had no efficacy in reducing TBI lethality supports this conclusion. It has been well documented that p53-dependent apoptosis of bone marrow cells contributes to the pathogenesis of radiation HP syndrome [5,23], and that the syndrome can be effectively suppressed by p53 inhibition [6,7,8,9,10]. Considering the fact that isorhamnetin did not strongly inhibit p53 activity and has no antiapoptotic effect, its ineffectiveness against bone marrow death may be somewhat reasonable.

In contrast to the ineffectiveness against TBI lethality, isorhamnetin successfully improved survival rate in mice after a lethal dose of SBI. Modulating the p53–p21 pathway is one of the key strategies for suppressing the GI syndrome. Compared with p53 wild-type mice, super-p53 mice with two more copies of Trp53 [24] are less sensitive to the GI injury [5], and p21 knockout mice are more sensitive to the injury [4,5]. The enhancement of p21 induction promotes cell cycle arrest, which may provide adequate time to repair radiation DNA damage [25]. We previously reported 5-chloro-8-quinolinol as a modulator of p53 transactivation that effectively inhibits the GI syndrome through the induction of p21. Others demonstrated similar results by using RG7112, a chemical compound that activates p53–p21 pathway through the inhibition of p53–Mdm2 interaction [26]. Considering the fact that isorhamnetin moderately inhibited p53–p21 pathway in irradiated MOLT-4 cells, it would exert the suppressive effect against GI death independent of p53.

Isorhamnetin showed slightly and markedly enhanced H2AX and ATM phosphorylation, respectively, in irradiated MOLT-4 cells, and an ATM-dependent radioprotective effect with increasing 53BP1 recruitment in Hep G2 cells, which may be associated with the suppressive effect of isorhamnetin against radiation GI death. 53BP1 is a principle component of DNA damage repair process and known to regulate the balance between homologous recombination and non-homologous end joining [27,28]. Activated ATM phosphorylates H2AX at Se139, which allows the recruitment of 53BP1 to the site of DNA double-strand breaks [29]. 53BP1-dificient mice have been shown to exhibit hypersensitivity to TBI of 7–8 Gy and succumb to GI and HP failures [30,31]. Furthermore, 53BP1 is important for maintaining euploidy and prevention of genome instability in the intestinal enterocytes of mice exposed to radiation [32]. Isorhamnetin may contribute to maintaining GI integrity by the upregulation of DNA damage repair through 53BP1, while the role of 53BP1 in the development of radiation GI syndrome is not well known. Detailed studies are further required to clarify the action mechanism of isorhamnetin.

In this study, isorhamnetin was found to have an ATM-dependent radioprotective benefit against radiation GI syndrome. Our data suggested that ATM-dependent reconvocation of 53BP1 is responsible for the mechanism of action of isorhamnetin. Its use may be useful for reducing GI toxicity in local exposure situations, such as lower abdominal radiotherapy.

## Data Availability

The data that support the findings of this study are available from the corresponding author, Akinori Morita, upon reasonable request.

## Supplementary Materials

The following are available online at https://www.mdpi.com/article/10.3390/genes12101514/s1, Figure S1: Screening of radioprotective flavonoids by WST-8 assay using MOLT-4 cells, Figure S2: Apigenin gave a higher survival rate to 24 Gy-SBI mice than the vehicle, but this difference did not reach statistical significance (*p* = 0.06).
