# Supplementary material for: Isorhamnetin Promotes 53BP1 Recruitment through the Enhancement of ATM Phosphorylation and Protects Mice from Radiation Gastrointestinal Syndrome
Source: Genes (Basel). 2021 Sep 26;12(10):1514. doi: 10.3390/genes12101514 (PMC8535534; doi:10.3390/genes12101514)
Supplement: Supplementary file 1 [file genes-12-01514-s001.zip › Supplementary figures.pdf]

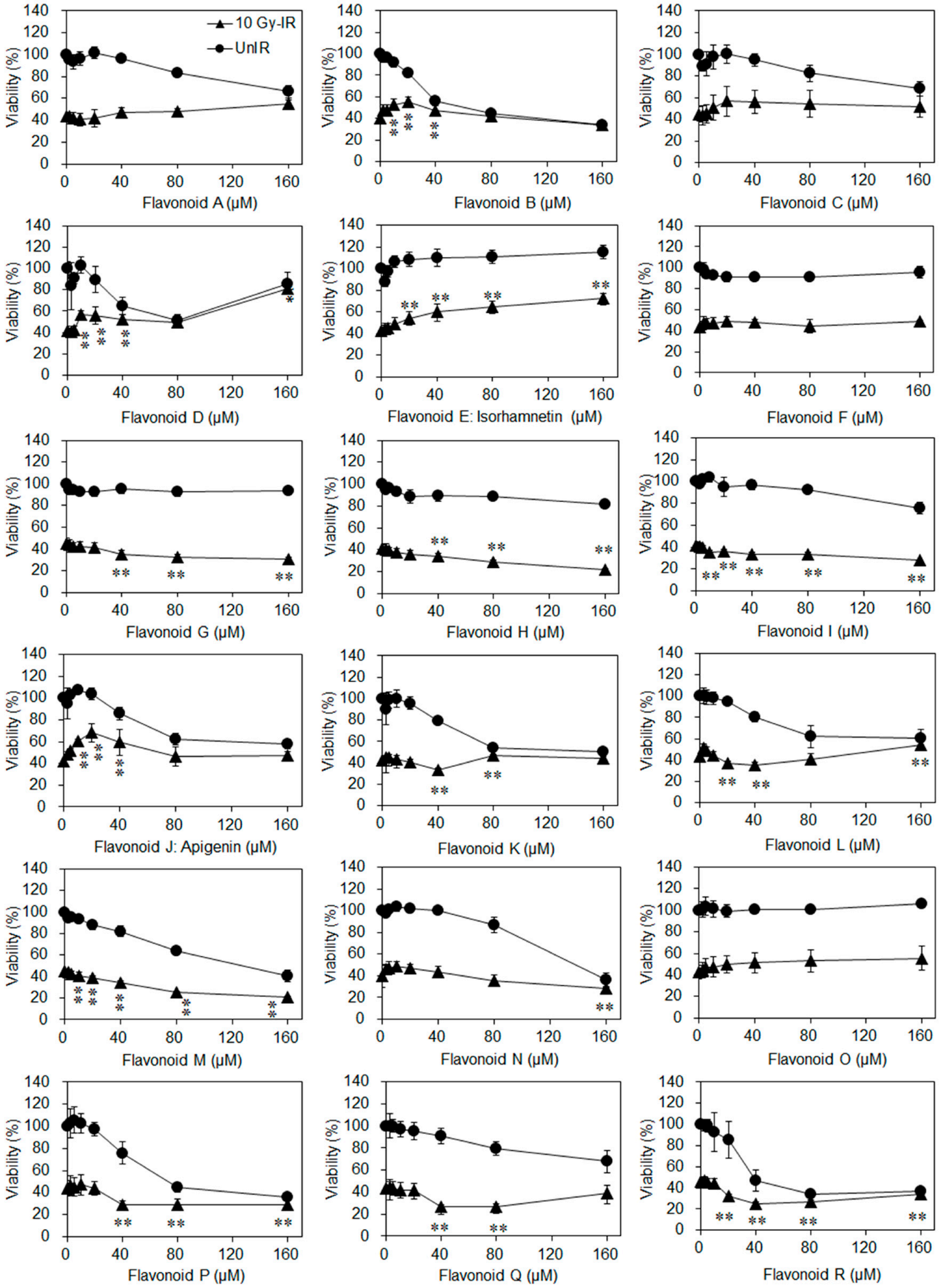

Supplementary Figure S1. Screening of radioprotective flavonoids by WST-8 assay using MOLT-4 cells. Eighteen kinds of flavonoids were examined in a blind manner. Each flavonoid was randomly selected, alphabetized (A to R), and added to the culture medium 1 hour before 10 Gy-irradiation. Viability of irradiated MOLT-4 cells was assessed 24 hours of post irradiation (n = 3).

Flavonoid B, D, E, J, K, and L significantly increased viability in irradiated cells at certain concentrations. Flavonoid E (isorhamnetin) and J (apigenin) were found as two of the most radioprotective flavonoids. Data represent the mean ± standard deviation. Dunnett's test was used for examine statistical differences in the viability. Asterisks indicate a significant difference (* $p < 0.05$ and ** $p < 0.01$) between groups. *IR* irradiated, *UnIR* unirradiated.

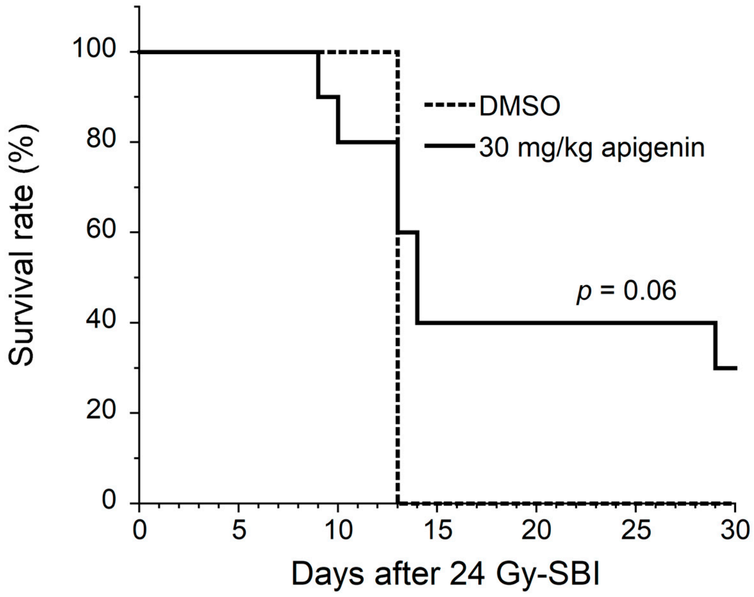

Supplementary Figure S2. Apigenin gave a higher survival rate to 24 Gy-SBI mice than the vehicle, but this difference did not reach statistical significance ($p = 0.06$). Female ICR mice (8 weeks of age, n = 10 in each group) were given a single intraperitoneal injection of 30 mg/kg apigenin or vehicle (10% DMSO in olive oil) 30 min before 24 Gy-SBI. *SBI* subtotal-body irradiation, *DMSO* dimethyl sulfoxide.
